# The northernmost Eurasian Miocene beavers: *Euroxenomys* (Castoridae, Mammalia) from Olkhon Island, Lake Baikal (Eastern Siberia)

**DOI:** 10.1007/s12549-022-00555-x

**Published:** 2022-11-23

**Authors:** Thomas Mörs, Signe Hägglund, Margarita A. Erbajeva, Nadezhda Alexeeva, Alexander A. Shchetnikov, Gudrun Daxner-Höck

**Affiliations:** 1 Department of Palaeobiology, Swedish Museum of Natural History, P.O. Box 50007, SE 10405 Stockholm, Sweden; 2 Bolin Centre for Climate Research, Stockholm University, Stockholm, Sweden; 3 Dobretsov Geological Institute, Siberian Branch, Russian Academy of Sciences, Sahianova Str. 6a, Ulan-Ude, 670047 Russia; 4 Geological Institute, Russian Academy of Sciences, Pyzhevsky lane 7, Moscow, 119017 Russia; 5 Institute of the Earth’s Crust, Siberian Branch, Russian Academy of Sciences, Lermontova str. 128, Irkutsk, 664033 Russia; 6 A.P. Vinogradov Institute of Geochemistry, Siberian Branch, Russian Academy of Sciences, Favorskogo str. 1a, Irkutsk, 664033 Russia; 7 Laboratory of Geoarchaeology of Baikal Siberia, Irkutsk State University, 5 Armii str. 52, Irkutsk, 664025 Russia; 8 Rupertusstr. 16, 5201 Seekirchen, Austria; 9 Natural History Museum Vienna, Burgring 7, 1010 Vienna, Austria

**Keywords:** East Asia, Morphometrics, Neogene, Orleanian, Rodentia, Systematics

## Abstract

The castorid dental material described in this paper derives from Miocene, fossiliferous deposits of the Baikal rift valley, exposed at Tagay Bay on Olkhon Island in the Lake Baikal, in eastern Siberia. It consists of maxillary fragments and isolated upper and lower teeth of the small trogontheriine beaver *Euroxenomys minutus* (von Meyer, 1838). It is the first record of the species in Asia and at the same time the northernmost occurrence of Eurasian Miocene beavers. The magnetostratigraphic correlation of the Tagay -1 section, indicates a late Burdigalian, Early/early Middle Miocene age of ~16.5 to ~16.3 Ma that corresponds to the Mammalian Neogene zone MN4/5. The presence of *E. minutus* in Tagay is an indicator for an Orleanian European-Siberian bioprovince during the Mid-Miocene Climate Optimum, and for a continuous belt of humid, warm-temperate to subtropical forests, stretching from Europe to Siberia, and probably further to East and South-Eastern Asia. In Eurasia, beaver remains are an indicator of permanent water bodies, which is in agreement with the palaeoenvironment of the Tagay locality.

## Introduction

The fossil record of Eurasian Castoridae during the Early and Middle Miocene stretches from Europe to East Asia. In Europe, the record during the Orleanian and Astaracian Land Mammal Ages/Mega-Zones, Mammalian Neogene Zones 3 to 8 (20.04 to 11.1 Ma), is particularly rich with many localities in Austria, Czech Republic, France, Germany, Greece, Poland, Portugal, Spain, Switzerland and Turkey (Hugueney 1999). From these localities the small-sized *Euroxenomys minutus*, *Steneofiber eseri* and ?*Dipoides* sp., medium-sized *Chalicomys jaegeri* and *Steneofiber depereti* and large-sized *Anchitheriomys suevicus* have been reported (Hugueney 1999). The northernmost European localities are Bełchatów B (MN 6) in central Poland (51°14′01″N 019°18′46″E) and Hambach 6C (MN 5) in northwestern Germany (50°54′39″N 6°30′10″E), from where the latter two species have been described (Hugueney 1999; Stefen and Mörs 2008; Mörs and Stefen 2010).

In East Asia, beavers are much less abundant during this time interval (Shanwangian and Tunggurian Land Mammal Stages/Ages (20.04 to 11.1 Ma). From localities in the Junggar, Linxia and Qaidam basins, in the Tunggur area in Inner Mongolia, in the coastal areas of eastern and northern China, in the Mae Moh and Chiang Muan basins in northern Thailand and in the Kani and Mizunami basins in Central Japan the small-sized *Euroxenomys nanus*, the medium-sized *Steneofiber siamensis*, *?Steneofiber changpeiensis, Hystricops mengensis* and *Monosaulax tungurensis,* the large-sized *Minocastor godai* and *Anchitheriomys tungurensis*, and the giant-sized *Youngofiber sinensis* have been reported (Suraprasit et al. 2011; Deng et al. 2013; Tomida et al. 2013; Mörs et al. 2016; Mörs and Tomida 2018; Qiu and Li 2016; Li et al. 2019).

The goal of our paper is to describe the first Miocene Castoridae from eastern Siberia, which also represent the northernmost record in East Asia.

## Geological setting of the Tagay locality

**Fig. 1.**
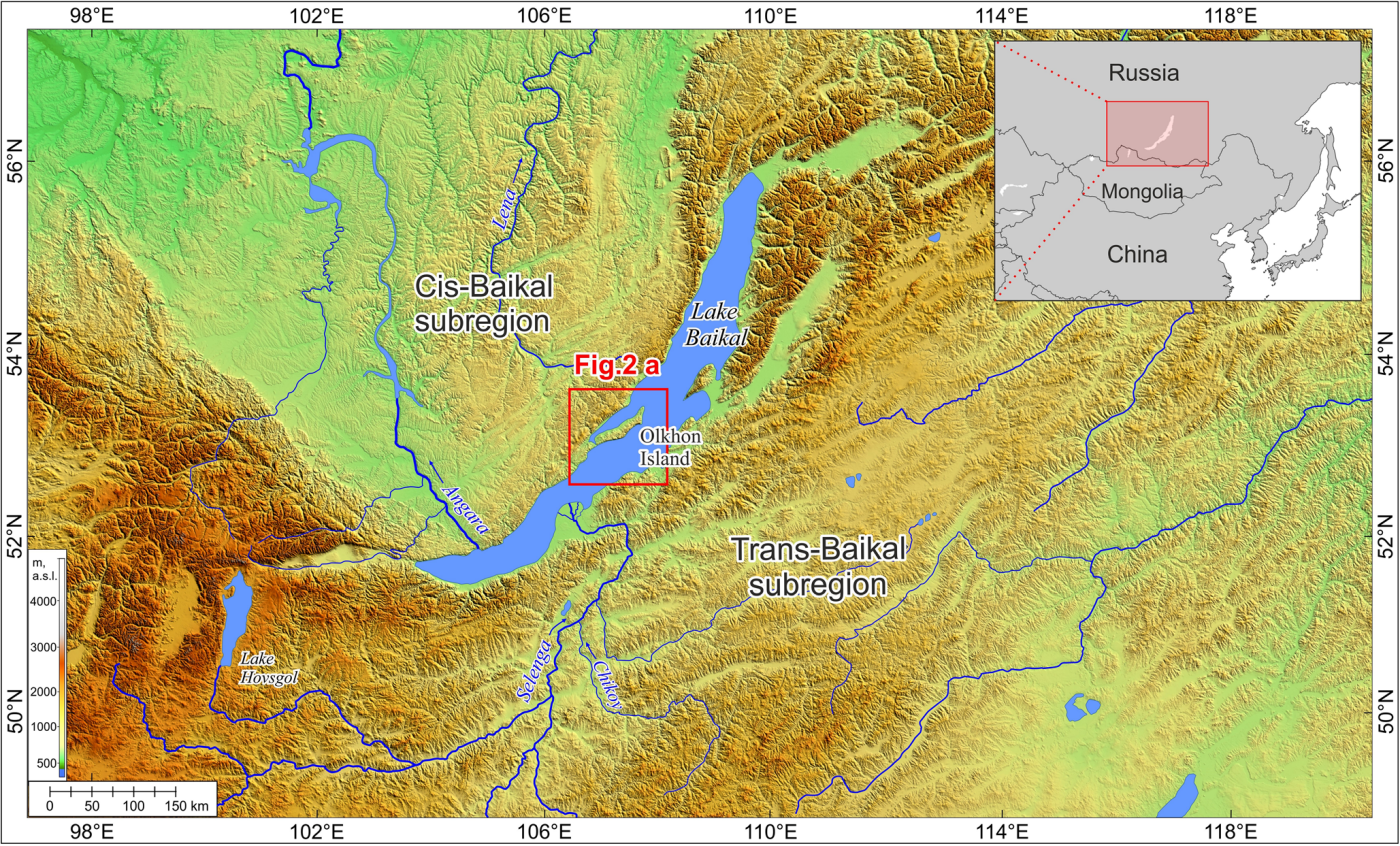
Map of the Baikal region and its position in Siberia (inset). The position of Olkhon Island is indicated by the red box (see also Fig. 2a). From Daxner-Höck et al. (2022a, this issue).

**Fig. 2.**
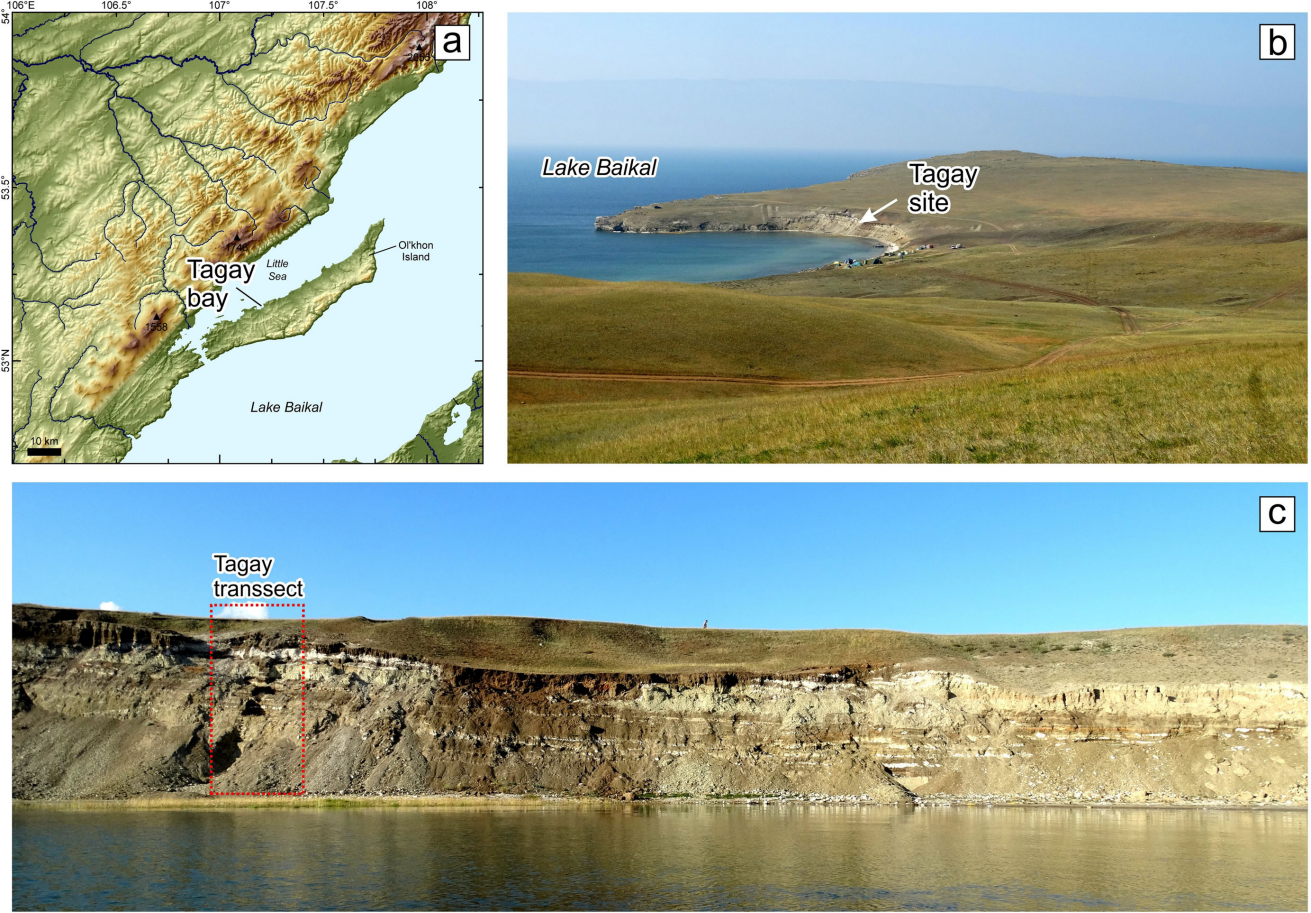
Map with the location of Tagay Bay (**a**), view from south-west to the Tagay site (**b**), and view from north to the Tagay transect (**c**). From Daxner-Höck et al. (2022a, this issue).

**Fig. 3.**
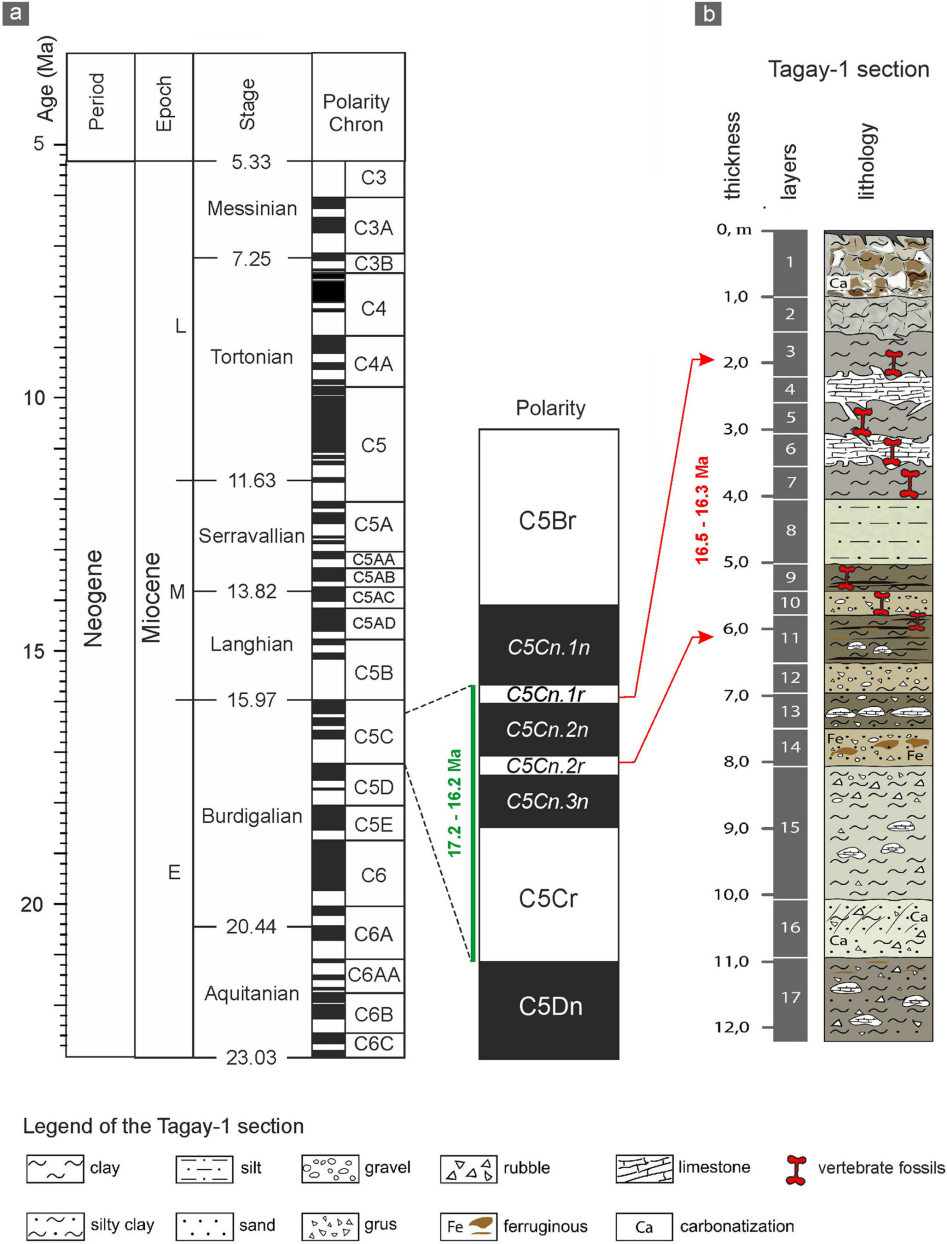
Correlation of the Magnetic Polarity Time Scale with the magnetic polarity pattern of the Tagay transect (**a**), and correlation with the vertebrate-bearing layers 11 to 3 of the lithological section (**b**). From Daxner-Höck et al. (2022d, this issue).

The Tagay section (53°9′34.74″N, 107°12′43.12″E) at Tagay bay is located on the northwestern coast of Olkhon Island in Lake Baikal (Figs 1, 2). Miocene sediments overlie here Paleozoic bedrock, mainly biotite gneisses and migmatites with deep pockets of a Cretaceous to Paleogene weathering crust of the ancient peneplain (Daxner-Höck et al. 2022d, this issue; Kazansky et al. 2022, this issue). The sedimentary environment of Tagay is characterised by debris flows of alluvial fans, floodplain accumulations and calcrete palaeosol horizons (Daxner-Höck et al. 2013; Daxner-Höck et al. 2022a, this issue; Ivanova et al. 2022, this issue). The sedimentary sequence of Tagay belongs to the Proto-Baikal stage, when small, shallow lakes and wetlands existed in the western part of the Baikal Depression (Daxner-Höck et al. 2022d, this issue). Palaeontological and palaeobotanical data indicate a broad array of palaeoenvironments, from shallow lakes and swamps, riparian forests with dense undergrowth, woodlands, to restricted steppe (Erbajeva and Alexeeva 2013; Daxner-Höck et al. 2013; Daxner-Höck et al. 2022d, this issue; Voyta et al. 2022, this issue). The Tagay small mammal fauna, apart from the beaver here described, consists of erinaceid, talpid, plesiosoricid and soricid insectivores, a palaeolagid lagomorph, and sciurid, aplodontid, mylagaulid, glirid, eomyid and cricetid rodents (Tesakov and Lopatin 2015; Daxner-Höck et al. 2013; Daxner-Höck et al. 2022a,b,c,d, this issue; Erbajeva et al. 2022, this issue; Voyta et al. 2022, this issue). Daxner-Höck et al. (2022d, this issue) state that the Tagay fauna biostratigraphically best correlates with the European Neogene Mammal Zones MN5, and with the Shanwangian Land Mammal Age/Stage. This correlates with the subchrons C5Cn.2r – C5Cn.1r of Chron C5C according to the magnetic polarity pattern of the upper part of the Tagay section (Kazansky et al. 2022, this issue), indicating a late Burdigalian age, ~16.5 to ~16.3 Ma (Daxner-Höck et al. 2022d, this issue) (Fig. 3).

## Material and methods

The material from the Tagay locality was partly surface collected in 1978, and partly excavated in 2014 (Fig. 2), when 17 layers were recognised in total, with seven vertebrate bearing layers (Fig. 3) of which layers 5, 7, 9 and 10 produced the castorid fossils described here. For detailed description of fieldwork methods, see Daxner-Höck et al. (2022b, this issue). The morphological description of the teeth follows Stirton (1935), and Stefen and Mörs (2008). Tooth measurements and drawings were taken/made using a Leica MZ6 discussion microscope equipped with an ocular micrometer and camera lucida. We measured the occlusal surface because most teeth are attached to jaw fragments. All measurements are given in mm.

The fossil material is stored in the collection of the Zoological Institute of Russian Academy of Sciences (ZIN), St. Petersburg, Russia.

**Anatomical abbreviations: dP4**: upper deciduous molar, **P4/p4**: upper and lower premolar, **M1/m1**: upper and lower first molar, **M2/m2**: upper and lower second molar, **m3**: lower third molar.

**Institutional abbreviations**: **ZIN**: collection of the Zoological Institute of Russian Academy of Sciences, St. Petersburg.

## Systematic palaeontology

Order Rodentia Bowdich, 1821

Family Castoridae Hemprich, 1820

Genus *Euroxenomys* Samson and Radulesco, 1973

**Fig. 4.**
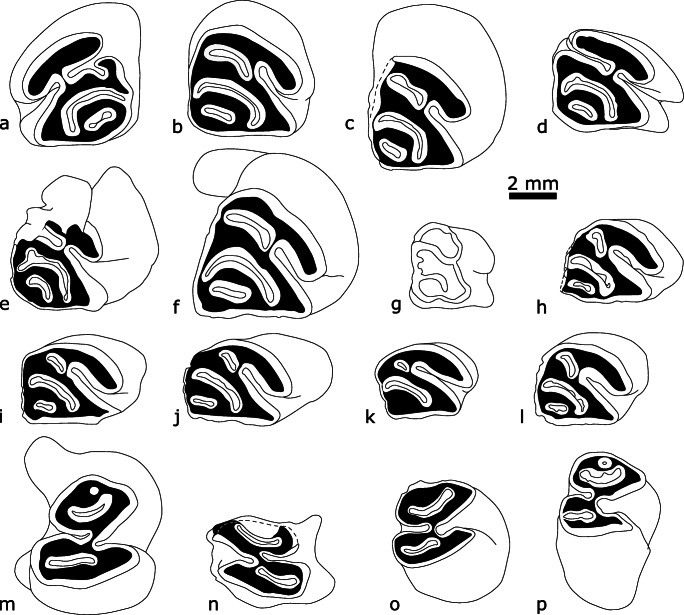
*Euroxenomys minutus* (von Meyer, 1838) from the Tagay locality on Olkhon Island, Lake Baikal, Siberia. **a** Left P4 (ZIN 106953); **b** right P4 (ZIN 106954); **c** right P4 (ZIN 106955); **d** right P4 (ZIN 106956); **e** right P4 (ZIN 106957); **f** right P4 (ZIN 106958); **g** right dP4 (ZIN 106959); **h** right M1 (ZIN 106960); **i** right M1/2 (ZIN 106961); **j** right M1/2 (ZIN 106962); **k** right M2 (ZIN 106954); **l** right M2 (ZIN 106960); **m** right p4 (ZIN 106963); **n** left m1/2 (ZIN 106965); **o** right m1/2 (ZIN 106964); **p** right m3 (ZIN 106966).

**Fig. 5.**
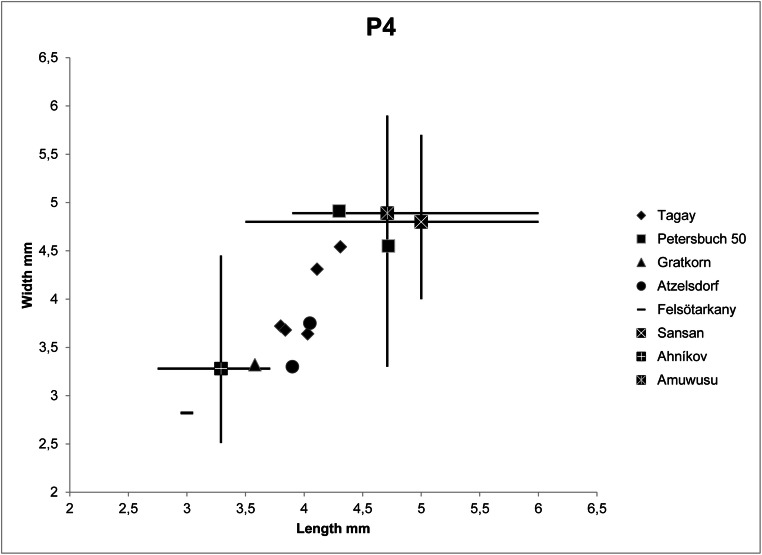
Length-width diagram of upper premolars of *Euroxenomys minutus* from Tagay, in comparison with *E. minutus* from other European localities, *Steneofiber* cf. *dehmi* from Ahnikov, Czech Republic, and *Monosaulax tungurensis* from Amuwusu, Inner Mongolia. For references, see text.

**Fig. 6.**
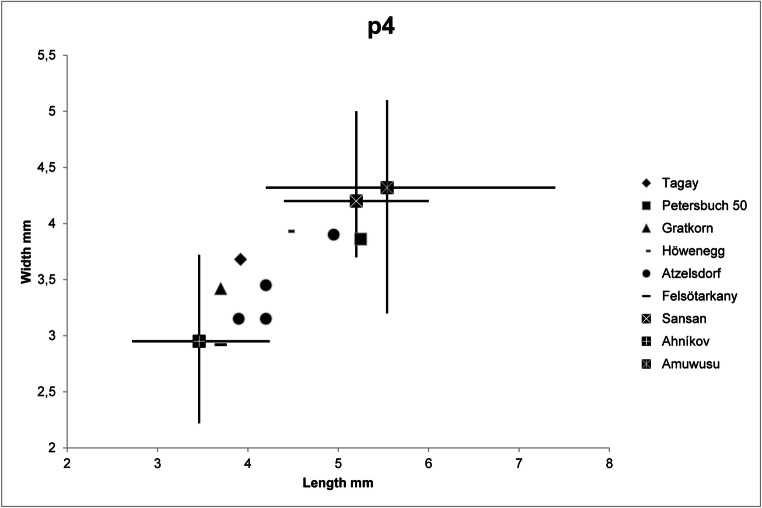
Length-width diagram of lower premolars of *Euroxenomys minutus* from Tagay, in comparison with *E. minutus* from other European localities, *Steneofiber* cf. *dehmi* from Ahnikov, Czech Republic, and *Monosaulax tungurensis* from Amuwusu, Inner Mongolia.. For references, see text.

*Euroxenomys minutus* (von Meyer, 1838) (Figs 4, 5 and 6, Table 1)

**Table 1.** Measurements (in mm) of *Euroxenomys minutus* teeth from Tagay (Olkhon Island, Baikal region, Siberia).

Inv.Number	Element	L/R	L	W
ZIN 106953	left P4	left	3.80	3.72
ZIN 106954	right P4	right	4.11	4.31
ZIN 106955	right P4	right	4.03	3.64
ZIN 106956	right P4	right	3.84	3.68
ZIN 106958	right P4	right	4.31	4.54
ZIN 106957	right P4	right		3.25
ZIN 106959	right dP4	right	2.90	2.35
ZIN 106960	right M1	right	2.86	3.64
ZIN 106954	right M2	right	2.78	3.60
ZIN 106960	right M2	right	2.94	3.52
ZIN 106961	right M1/2	right	2.82	3.41
ZIN 106962	right M1/2	right	2.86	3.68
ZIN 106963	right p4	right	3.92	3.68
ZIN 106964	right m1/2	right	3.09	3.05
ZIN 106966	right m3	right	2.90	2.62

1964 *Monosaulax* – Logachev, Lomonosova and Klimanova: 41.

2013 *Monosaulax* sp. – Erbajeva and Alexeeva: 501.

**Locality, Stratigraphy:** Tagay Bay, layers 5, 7, 9 and 10 of the Tagay section; Olkhon Island (Baikal Lake), Irkutsk Region; Eastern Siberia; Tagay Formation; Early/Middle Miocene transition, MN5.

**Material:** left maxillary fragment with P4 (ZIN 106953), right maxillary fragment with P4, M2 (ZIN 106954), right maxillary fragment with P4 (ZIN 106955), right maxillary fragment with M1-2 (ZIN 106960); isolated teeth: right P4 (ZIN 106956), right P4 (ZIN 106957), right P4 (ZIN 106958), right DP4 tooth germ (ZIN 106959) (2014, layer 10), right M1/2 (ZIN 106961), right M1/2 (ZIN 106962), right p4 (ZIN 106963) (2014, layer 7), right m1/2 (ZIN 106964), left m1/2 fragment (ZIN 106965) (1978), right m3 (ZIN 106966) (2014, layer 5), seven I fragments (ZIN 106967) (2014, layer 9).

**Measurements** (in mm): See Table 1.

**Description:** The upper dentition is represented by four maxillary fragments with P4, P4 and M2, and M1-M2 in situ; additionally there are isolated teeth: one DP4 tooth germ, three P4 and two M1/2; there is no M3 preserved. One of the P4 (ZIN 106958) and the two M1/2 (ZIN 106961, 106962) from Layer 9 most likely represent one individual, based on preservation and tooth wear. The upper P4 is the largest of all cheek tooth positions, accordingly the upper premolars are significantly larger than M1/2. In contrast to the upper dentition, the lower dentition is represented by isolated teeth only: one p4, two m1/2, and one m3. The premolar is the largest of the lower cheek teeth. Most of the cheek teeth are considerably worn. In some flexi(-ds)/fossettes(-ids)/striae(-iids) there is little tooth cementum preserved, but it seems that there were no massive cementum fillings.

**DP4 tooth germ** (Fig. 4g): This completely unworn tooth germ is very low-crowned, with the labial side much lower than the lingual one. In occlusal view, the shape is close to rectangular, although anterolingually rounded. Proto-, para- and metacone are clearly visible. The hypostria runs down to the crown base where it closes. Para-, meso- and metaflexus show only shallow openings on the labial side. The paraflexus occupies ¾ of the anterior tooth width, the long mesoflexus is bent and ends at the posterolingual corner, and the short metaflexus has a shallow connection to the posterior part of the mesoflexus. There are no roots preserved, probably they were not yet formed in this ontogenetic stage.

**P4** (Fig. 4a-f): All six upper premolars are very similar in morphology, with the occlusal surface resembling a circular triangle with the anterior side rounded and a “pinched” lingual side (shorter than the labial side). Hypoflexus, paraflexus/-fossette, mesoflexus/-fossette and metafossette are discernible elements in all P4. In all premolars, the paraflexus/-fossette meets the hypoflexus, which is straight and directed oblique anteriorly. The long mesoflexus/-fossette is strongly bent and ends lingually at the posterior side, thus enclosing the short, mostly straight metafossette. The crown height, indicated by the enamel-covered crown, differs significantly from the labial to the lingual side, with being highest at the anterolingual side. Judging from a slightly worn, isolated P4 (ZIN 106956) the hypostria runs down half the crown height. The enamel band of the anteroloph and protoloph is thicker than the rest of the enamel band. The enamel surface is slightly wrinkled, which is best visible on the anterolingual side of the teeth. All premolars show an interdental wear facet on the posterior wall. The premolars show two roots, one massive, halfmoon shaped anterolingual one, and a shorter small, round posterolabial root. Two of the P4 (ZIN 106953, 106956) are only slightly worn (sensu Stefen and Mörs 2008), thus showing a labially open paraflexus. In ZIN 106953, the paraflexus shows an irregular posterior protrusion, whereas it is straight in ZIN 106956. The latter premolar has the longer parastria, but due to the mesofossette no mesostria, whereas in ZIN 106953 the mesoflexus is still slightly labially open, resulting in a very short mesostria. Three premolars (ZIN 106954, 106955, 106958) are medium worn (sensu Stefen and Mörs 2008), meaning that there is only the hypoflexus and -stria on the labial side. One P4 (ZIN 106957) shows an anterior protrusion in the mesofossette, and a slightly curved metafossette.

**M1/2** (Fig. 4h-l): All five upper first and second molars are very similar in morphology, resembling the P4. The occlusal surface is roughly kidney-shaped, shorter than P4. Hypoflexus, parafossette, mesofossette and metafossette are discernible elements in all molars with the exception of a heavily worn M2 (ZIN 106954) where the metafossette is missing. In all molars, the parafossette is shorter and more oblique than in P4. It meets the hypoflexus, which is straight and directed oblique anteriorly like in P4. The long mesofossette is only slightly curved in comparison to P4 and ends lingually at the posterior side. The short metafossette is straight and runs parallel to the posterior wall. The molars are clearly less high-crowned than the P4, but crown height differs in a similar way from the labial to the lingual side, with being highest at the anterolingual side. The hypostria runs down half the crown height. The enamel band of the anteroloph and protoloph is thicker than the rest of the enamel band. The molars show three roots, one massive, halfmoon shaped lingual one, and two small, short round labial roots. The M1 and M2 from the maxillary fragment (ZIN 106960) are very similar in their dental pattern, only the hypostria is slightly shorter in the M1. The same applies for the two M1/2 that supposedly represent a single individual, meaning that the molar with the shorter hypostria (ZIN 106962) would represent a M1, and the other tooth (ZIN 106961) a M2. The maxillary fragment ZIN 106961 exhibits the most worn M2 of the studied sample; the crown enamel on the labial wall is very low, the metafossette is worn away, instead the pulpa cave of the posterolabial root is shimmering through the dentine.

**p4** (Fig. 4m): The occlusal surface of the single lower premolar is shaped like a figure eight, with the posterior portion being wider than the anterior one. The anteroconid represents the anteriormost edge and is situated in the median line of the tooth. It is the highest area of the slightly convex occlusal surface. Hypoflexid, the remnant of an additional anterior fossettid, parafossettid, mesoflexid and metafossettid are discernible elements in this medium worn premolar (sensu Stefen and Mörs 2008). The parafossettid is curved and comes labially close to the anterior wall. Here, slightly detached to the midline, is also situated the additional anterior fossettid. The mesoflexid is transversely oriented, the hypoflexid is curved posteriorly. The straight metafossettid is transversely oriented and reaches the midline of the tooth. The mesostriid runs down half the crown height. The hypostriid is much longer and runs down ¾ of the crown height. The tooth crown is significantly higher on the labial wall, as indiated by the enamel cover. The enamel band is thickest on the posterior wall and on the anterior wall of the hypoflexid. The premolar has two roots of the same length, one anterior and one posterior. Both roots are half-moon shaped with the anterior one more curved and slightly more massive.

**m1/2** (Fig. 4n, o): The two m1/2 resemble morphologically the p4, but the occlusal surface is shorter, with a transverse anterior wall, thus they are nearly square in outline. Hypoflexid, parafossettid, mesoflexid and metafossettid are discernible elements. The hypoflexid is posteriorly-oblique oriented and ends at the median line of the teeth between mesoflexid and metafossettid. The oblique, long parafossettid is curved and in median position. The shorter metafossettid is less curved and parallel to the posterior wall of the teeth. The molars are clearly less high-crowned than the p4. The crown height is highest at the posterior side and lowest at the anterior side. The mesostriid is short, the hypostriid much longer and runs down ¾ of the crown height. As in the p4, the enamel band is thickest on the posterior wall and on the anterior wall of the hypoflexid. The molars show three roots, one transverse posterior one, and two small, short round anterior roots in the anterolingual and -labial corners.

**m3** (Fig. 4p): The molar is only slightly worn as indicated by an additional anterior fossettid. Tooth morphology is similar to the m1/2 but the crown height is clearly shorter than that of the m1/2. The tooth shows a narrowing in the distal half in occlusal and posterior views and there is no interdental wear facet present on the posterior wall.

## Comparisons

The specimens described above exhibit a number of diagnostic features that are characteristic of *Euroxenomys minutus,* according to Hugueney (1999) and Daxner-Höck and Bernor (2009); especially small size, the subtriangular P4 that is substantially bigger than the upper molars, the p4 being markedly larger than the lower molars, parallel oriented flexids/fossettids and hypostriae/-ids that do not reach the base of the crown. Another characteristic feature is the pronounced anteroconid of the p4 that forms an anteriormost edge (Fig. 4m). Unfortunately, the characteristic M3 is not present in the Tagay material. The occlusal pattern fits well with *E. minutus* from European Middle and Late Miocene localities, e.g. Sansan (MN 6) in France (Hugueney and Duranthon 2012), Gratkorn and Matatschen (MN 7/8) and Atzelsdorf (MN 9) in Austria (Daxner-Höck 2004; Daxner-Höck and Bernor 2009; Prieto et al. 2014), Anwil (MN 7/8) in Switzerland (Engesser 1972), Felsőtárkány (MN 7/8) in Hungary (Hir 2004) and Höwenegg (MN9) in Germany (Giersch et al. 2010). An additional anterior fossettid is as well preserved in juvenile teeth from Sansan and Atzelsdorf (op. cit.).

Metrically, the P4 from Tagay fit well with *E. minutus* from Sansan, the German MN 8 locality Petersbuch 50 (Stefen and Rummel 2003), Gratkorn and Atzelsdorf, although the largest Tagay P4 are in the lower range of Sansan premolars, and slightly smaller than the Petersbuch 50 specimens (Fig. 5). On the other hand all Tagay premolars are larger than the one from Felsőtárkány. P4 dimensions of the Early Miocene, morphologically different *Steneofiber* aff. *dehmi* from Ahnikov (MN 3) in Czech Republik (Šmejkal 2018) overlap with *E. minutus* (Fig. 5). The single lower p4 from Tagay fits well with premolars from Gratkorn, Atzelsdorf and Felsőtárkány, but is clearly smaller than all p4 from Sansan, and single premolars from Petersbuch 50, Atzelsdorf and Höwenegg (Fig. 6). As in the upper premolars, dimensions of p4 of *Steneofiber* aff. *dehmi* from Ahnikov overlap with *E. minutus* (Fig. 6).

When compared with the Asian Early/Middle Miocene castorid taxa, *E. minutus* from Tagay is similar in dental morphology to *Euroxenomys nanus* but larger than this Early Miocene *Euroxenomys* species (Mörs and Tomida 2018). *E. minutus* from Tagay is smaller than *Minocastor godai, Anchitheriomys tungurensis, Youngofiber sinensis*, *Steneofiber siamensis, ?Steneofiber changpeiensis, Hystricops mengensis, Eucastor plionicus* and *Monosaulax tungurensis*, although in the latter there is some overlap in size in the upper premolars from Amuwusu in Inner Mongolia (Fig. 5) (Suraprasit et al. 2011; Deng et al. 2013; Tomida et al. 2013; Mörs et al. 2016; Qiu and Li 2016; Li et al. 2019).

## Discussion

*Euroxenomys minutus* (von Meyer, 1838) from Tagay-1 is the first record of this small trogontheriine castorid in Asia, other-wise it is only known from Europe (including Turkey). *E. minutus* is a rare faunal element in Early and early Middle Miocene (MN 4 – MN 5) localities, and a common beaver in Middle and Late Miocene (MN 6 – MN 11) sites (Hugueney 1999). There is a single record of a second, smaller Eurasian *Euroxenomys* species, *Euroxenomys nanus* from the Early Miocene (equivalent to MN 3, Shanwangian) of Japan, which up to now represented the only record of the genus in Asia (Mörs and Tomida 2018). In North America, two species have been described, the *Euroxenomys wilsoni* from the Early Miocene (early Hemingfordian) of Colorado and Nebraska (Korth 2001, 2004) and *Euroxenomys inconnexus* from the Middle Miocene (early Barstovian) of Montana (Sutton and Korth 1995), but the New World fossil record of the genus is very scarce. The entire fossil record of Eurasian *Euroxenomys* ranges from the Early Miocene (Shanwangian/Orleanian, MN3) to the Late Miocene (Turolian, MN13). The magnetostratigraphic correlation of the vertebrate-bearing, upper part of the Tagay-1 section with the subchrons C5Cn.2r – C5Cn.1r of Chron C5C, indicates a late Burdigalian, Early/early Middle Miocene age, ~16.5 to ~16.3 Ma that can be correlated with MN4/5 (Fig. 3) (Daxner-Höck et al. 2022d, this issue; Kazansky et al. 2022, this issue).

The Tagay *Euroxenomys* represents also the northernmost occurrence of Eurasian Miocene beavers, given the site’s lati-tude of 53°9′34.74″N.

The presence of *E. minutus* in the Tagay fauna corroborates the close affinities with European Orleanian faunas, which has been pointed out earlier for the large mammals by Vislobokova (1994, 2004), for the snakes by Rage and Danilov (2008) and for the rodents by Daxner-Höck et al. (2013), and which has been recently confirmed for the insectivores by Voyta et al. (2022, this issue) and with new data for the rodents by Daxner-Höck et al. (2022a,b,c, this issue). This strongly indicates an Orleanian united European-Siberian palaeozoographical subregion as proposed already by Erbajeva and Alexeeva (2013). Such a bioprovince during the late Burdigalian (MN4/5) would require a continuous forest belt, stretching from Europe to Siberia, and probably further to East and South-Eastern Asia, as discussed earlier by, e.g. Pinecker and Mörs (2011). Warm-temperate, subtropical climate conditions in western and eastern Siberia during the Mid-Miocene Climate Optimum (Steinthorsdottir et al. 2021) were suitable for humid, polydominant broad-leaved forests with lignite-forming Taxodiaceae, some subtropical elements, e.g. Juglandaceae, *Liquidambar, Nyssa* and *Magnolia*, in combination with summer-green beeches und oaks (Arkhipov et al. 2005; Mashchuk and Akulov 2012; Erbajeva and Alexeeva 2013; Al Hamoud et al. 2019).

The beaver *E. minutus* indicates the presence of permanent water bodies, which is in general agreement with the palaeoenvironment reconstructed for the Tagay locality (Erbajeva and Alexeeva 2013; Daxner-Höck et al. 2013; Daxner-Höck et al. 2022d, this issue; Voyta et al. 2022, this issue).

## Conclusions

- The castorid remains from Tagay can be attributed to a single beaver species, the small-sized *Euroxenomys minutus* (von Meyer, 1838).
- The Tagay specimens represent the first record of the species in Asia and at the same time the northernmost occurrence of Eurasian Miocene beavers.
- The magnetostratigraphic correlation of the fossiliferous part of the Tagay transect, indicates an Early/early Middle Miocene age of ~16.5 to ~16.3 Ma.
- The record of a European castorid species in the Baikal rift is another indicator for an Orleanian European-Siberian bioprovince during the late Burdigalian (MN4/5), and for a continuous belt of humid, warm-temperate to subtropical forests, stretching from Europe to Siberia, and probably further to East and South-Eastern Asia during the Mid-Miocene Climate Optimum.
- The beaver remains indicate the presence of permanent water bodies, which is in agreement with the palaeoenvironment of the Tagay locality.

## Data Availability

All data generated or analysed during this study are included in this published article.
